# Flexibly funding WHO? An analysis of its donors’ voluntary contributions

**DOI:** 10.1136/bmjgh-2022-011232

**Published:** 2023-04-06

**Authors:** Obichukwu Iwunna, Jonathan Kennedy, Andrew Harmer

**Affiliations:** 1 Accident and Emergency Department, Mid Cheshire Hospitals NHS Foundation Trust, Crewe, UK; 2 Barts and The London School of Medicine and Dentistry, Wolfson Institute of Population Health, Centre for Public Health and Policy, Queen Mary University of London, London, UK

**Keywords:** public health, health policy

## Abstract

**Introduction:**

Since the 1970s, voluntary contributions have become an increasingly important component of WHO’s budget. As voluntary contributions tend to be earmarked for donor-specified programmes and projects, there are concerns that this trend has diverted focus away from WHO’s strategic priorities, made coordination and attaining coherence more difficult, undermined WHO’s democratic structures and given undue power to a handful of wealthy donors. In the past few years, the WHO Secretariat has pushed for donors to increase the amount of flexible funding they provide.

**Methods:**

This paper aims to add to the literature on WHO financing by constructing and analysing a dataset based on figures extracted from WHO documents for the period 2010–21. It aims to answer two questions: who funds WHO and how flexible is that funding?

**Results:**

Our analysis demonstrates that in the last decade voluntary contributions have steadily increased as a proportion of WHO’s budget, from 75% at the start of the period to 88% at the end. High-income countries and donors based in high-income countries provided 90% of voluntary contributions in 2020. Surprisingly, the share of voluntary contributions provided by upper middle-income countries was consistently less than the share by lower middle-income countries. Furthermore, in terms of their share of voluntary contributions, we found that upper middle-income countries contributed the least proportion of their gross national income to WHO.

**Conclusion:**

We conclude that WHO remains constrained by the conditions attached to the vast majority of funding that it receives from its donors. Further work on how to flexibly fund WHO is required. We recommend that the Agile Member States Task Group on Strengthening WHO’s Budgetary, Programmatic and Financing Governance continues the work of the Working Group on Sustainable Financing by focusing on the incentives that determine donor support for specified and flexible voluntary contributions.

**WHAT IS ALREADY KNOWN ON THIS TOPIC**

- Summarise the state of scientific knowledge on this subject before you did your study and why this study needed to be done.
- There are very few studies that focus on WHO funding and none this century that quantify the extent of voluntary contributions (VCs) to WHO, to the best of our knowledge.
- Quantifying donors’ voluntary donations contributes to ongoing debate about the extent to which WHO funding is earmarked and the impact this has on the Organisation’s ability to fulfil its mandate.

**WHAT THIS STUDY ADDS**

- Summarise what we now know as a result of this study that we did not know before.
- Our study confirms the existence of a ‘structural defect’ underpinning WHO’s funding model.
- VCs accounted for 75% of WHO’s budget in 2010 and rose to 88% by 2021.
- This last figure is 10% higher than the figure of four-fifths which is cited by WHO on its webpages and in the scholarly literature.
- High-income countries and donors based in high-income countries provided 90% of VCs.
- Lower middle-income countries provide more voluntary funding, both in relative and absolute terms, than upper middle-income countries.

**HOW THIS STUDY MIGHT AFFECT RESEARCH, PRACTICE OR POLICY**

- Summarise the implications of this study.
- We are not the first researchers to raise concerns about the impact of WHO’s reliance on VCs from a handful of donors based mostly in high-income countries.
- We demonstrate that over the last 10 years this reliance on VCs has grown markedly and by considerably more than is generally acknowledged.
- This is likely to have further undermined WHO’s ability to fulfil its mandate, set out in its constitution, to ‘act as the directing and coordinating authority on international health work’ within the United Nations system.
- Our study underlines the pressing need for thorough reforms to the way WHO is financed.

## Introduction

WHO receives two main forms of financial contributions. The assessed contributions (ACs) it gets from member states are fully flexible, meaning WHO can decide how that money is spent. According to WHO, ACs ‘are a key source of financing for the Organisation, providing predictable financing, helping to minimise the dependence on a narrow donor base and allowing resources to be aligned to the Programme Budget’.^1^ In contrast, voluntary contributions (VCs) that WHO receives from member states and non-state actors from both the profit and not-for-profit sectors are overwhelmingly earmarked, that is, they come with conditions attached that specify the programmes and projects that the donor will fund (box 1).ACs are sometimes referred to as ‘regular budget funds’ and VCs as ‘extra-budgetary funds’. The latter are governed by Article 57 of WHO’s Constitution. WHO also receives in-kind or in-service contributions such as medical supplies and contractual services, although these account for a small proportion of the total budget.

Box 1 **WHO funding streams**

**Assessed contributions (ACs)**

ACs are fully flexible, compulsory dues remitted by individual member states as a single transaction, usually through their health ministries or departments. The amount to be paid is determined by eight criteria, which include a country’s gross national income, debt burden and per capita income. There are minimum and maximum assessment rates.^45^ The USA will contribute US$219.3 million in ACs for the 2022–23 biennium whereas Belize will contribute US$9970.

**Voluntary contributions (VCs)**

VCs to WHO are non-compulsory, non-assessed funds received from member states, and non-state donors that include individuals, private and public organisations. They can be divided into three main subcategories:

**Specified VCs**

This money must be spent on priorities, projects and programmes earmarked by the donor. They are not flexible. While most VCs are specified, a small but increasing amount of VCs include a degree of flexibility.

**Core VCs**

These voluntary funds are provided by donors to fund the Programme Budget as a whole. They sit within the Core Voluntary Contributions Account of the General Fund. They are not tied to any specific projects or purposes and are described by WHO as fully or highly flexible.

**Thematic VCs**

Also known as ‘thematic and strategic engagement funds’ or ‘voluntary contributions—core’, they are provided by a small but increasing number of member states. They are directed towards specific health themes, such as universal health coverage, polio eradication or governance of WHO. Because they include ‘funds that are earmarked for purposes within the Programme Budget’, they are considered partially or of a medium level of flexibility.^35^

In the 1970s, ACs accounted for three-quarters of WHO’s revenues. In the early 1980s, the World Health Assembly (WHA) applied a ‘zero real growth policy’ to WHO’s regular budget, meaning that ACs were frozen except for adjustments to take account of inflation. Later, in 1993, the WHA introduced a ‘zero nominal growth’ policy which removed the inflation adjustment.^2^ Consequently, ACs began to fall in real terms and WHO became increasingly reliant on VCs. By the early 1990s, VCs overtook ACs as WHO’s main source of funding. Since 2000, as WHO notes, ‘assessed contributions have declined as an overall percentage of the Programme Budget and have, for several years, accounted for <20% of the Organisation’s financing’.^1^

Numerous reports and analysis from WHO and by independent researchers have highlighted concerns with the way that the Organisation is funded.^3–5^ Most recently, M’ikanatha and Welliver have suggested that there are ‘structural defects’ supporting WHO’s funding model that compromise the Organisation’s legitimacy.^6^ According to the authors, the three main elements of the structural defect are: ‘WHO’s inadequate level of financing; lack of direct control over 80% of its funds; and unbalanced participation, such that over 60% of financing originates from only nine donors’.[M’ikanatha,^6^ p4] One worry relates to the size of WHO’s budget, which is seen as being far too small for it to perform its broad mandate to ‘act as the directing and coordinating authority on international health work’ within the United Nations (UN) system.^7^ Reddy *et al* observed in 2018 that WHO’s 2018–19 budget of US$4.4 billion was approximately equal to the University Hospital in Geneva.^2^ We would add that it also compared unfavourably with the budgets of the US Centers for Disease Control and Prevention (US$6.5 billion), the UK’s Public Health England (recently renamed UK Health Security Agency) (US$5.24 billion) and the Global Fund to fight AIDS, Tuberculosis and Malaria (US$3.9 billion). It was only fractionally higher than the total operating expenditure of Barts and the London NHS Trust (£1.6 billion/US$2.1 billion), which is just one of over 200 such trusts in the UK.^8^

Another worry relates to the dominant role that VCs now play in funding UN Organisations.^9^ Earmarking transfers power from WHO to donors because it allows donors to specify how their contributions are spent. It therefore has the potential to divert WHO’s focus away from its core priorities, undermine the ostensibly democratic structures of the WHA and make coordination and attaining coherence more difficult.^10 11^ The consequences of earmarked, VCs extend beyond their impact on individual international organisations and can threaten the legitimacy of multilateralism as a mode of international relations.^12 13^ Some VCs are more flexible than others (box 1) and there has been a small increase in flexible and thematic VCs as a % of VCs to the General Fund (from 8% to 14%) since 2020.^14^ VCs are also by definition much less predictable, which further complicates WHO budget planning. In recent years, three intersecting factors have heightened interest in the issue of flexible funding and its impact on the functioning of WHO.

First, regarding polio transition planning, scholars have described the financial implications of winding down the Global Polio Eradication Initiative (GPEI).^15^ At the WHA in May 2017, member states noted ‘with great concern’ the reliance of WHO on funding from the GPEI and requested the Director General (DG) to develop a strategic action plan to ‘mitigate the possible impact on the ramp-down of (GPEI) on the long-term sustainability of (WHO’s) key assets’.[WHO,^16^ p2] A summary of the mid-term evaluation of the Strategic Action Plan on Polio Transition, which was presented to member states at the 75th WHA (WHA75) in May 2022, noted: ‘sustainability, to a large degree, hinges on securing flexible and predictable financing for a continued polio transition response. Fragmented and unpredictable funding are major issues affecting planning for integration and transition’. [WHO,^17^ p11]

Second, a number of high-level reports and reviews have focused attention on the need for increased flexibility of WHO funding.^18 19^ These documents informed a period of consultation during 2021–22 led by WHO’s Working Group on Sustainable Financing (WGSF) whose final report was approved by member states at WHA75.^20^ The report recommended 50% of the base segment of the 2022–23 programme budget be funded by ACs by 2030. This is significantly less than 66% recommended by the Independent Panel on Pandemic Preparedness and Response (IPPPR) or up to 100% suggested as possible by The Independent Expert Oversight Advisory Committee.^21^ The WGSF final report also supported the IPPPR recommendation for a replenishment mechanism open to state and non-state donors, and which could potentially provide greater flexibility for funding to the base segment of the programme budget. These reforms are ongoing at the time of writing and will be taken forward by The Agile Member States Task Group on Strengthening WHO’s Budgetary, Programmatic and Financing Governance (Task Group).

The base segment of the programme budget is important because it covers ‘work done across all three strategic priorities (Universal Health Coverage, Health Emergencies and Health and Well-being) as well as the enabling functions—by country offices, regional office and headquarters’.^22^ These programmes reflect the core mandate of WHO and constitute “the largest part of the proposed Programme Budget in terms of strategic priority-setting, detail and budget figures’. Furthermore, unlike activities funded by earmarked contributions, ‘WHO has exclusive strategic and operational control over the activities concerned and over the choice of means, the location and the timing of implementation’.[WHO,^23^ p15]

Finally, the COVID-19 pandemic resulted in unprecedented additional funding to WHO: total contributions exceeded the approved 2020–21 Programme Budget of US$5.8 billion by approximately US$2.2 billion.^14^ However, none of the additional funding for the 2020–21 period was directed to the base programme segment of WHO’s budget: Polio Eradication, and Emergency Operations and Appeals segments were the main beneficiaries.

Despite heightened interest in VCs and their impact on the functioning of WHO, the topic has received comparatively little attention in peer-reviewed journals.^3 6 24 25^ This paper aims to add to the literature by constructing and analysing a dataset based on WHO figures for the period 2010–21 in order to answer two questions: who funds WHO and how flexible is that funding?

## Methods

This paper used publicly available WHO documents to construct a dataset to analyse the Organisation’s funding for the period 2010–21. For AC figures, we consulted WHO’s ACs website, specifically its annual ‘Status of Collection’ reports.^26^ ACs are approved at the WHA and are expected to be paid annually on 1 January each year (box 2). Due to currency fluctuations, the actual value of ACs paid to WHO can differ from the budgeted figure. In 2021, for example, member states’ annual assessment amounted to US$488 million. However, an exchange rate gain of US$38 million and other adjustments increased the annual assessment to US$549 million.^17^ We therefore distinguish between ACs approved at the WHA and ACs received, using the former in our analysis. In the ‘Results’ section, we express ACs as a % of the approved Programme Budget and as a % of the final Programme Budget. By ‘final Programme Budget’ we mean the amount of money that is eventually directed to the Programme Budget (box 2). For example, for the 2020–21 biennium, the approved budget was US$5840 million but the final amount eventually received was US$8004 million (online supplemental file 1). Providing both data allow a comparison of ACs as a % of the anticipated budget for each biennium against the total revenue actually received.

10.1136/bmjgh-2022-011232.supp1 Supplementary data

Box 2 **Glossary of budget terminology**

**Programme Budget**

The revenue that WHO receives from assessed and voluntary contributions from member states and donors which funds its biennial Programme Budget. The WHO Secretariat presents an estimated budget to the World Health Assembly for approval.

**Approved and Final Programme Budget**

The approved Programme Budget is the budget approved by WHO’s member states at the World Health Assembly, which is held in May each year. Typically, it is far less than the total donations eventually received by WHO for the biennium. This is because of the unpredictable nature of VCs (both in terms of quantity and also when they are received) as well as external global health events. We therefore distinguish between the *approved* and what we refer to in our analysis as the *final* Programme Budget.

**Programme Budget Segments**

The Programme Budget comprises four budget segments: Base Programmes; Emergency Operations and Appeals; Polio Eradication and Special Programmes. The Base Programmes segment (often referred to as the ‘base budget’) is the largest segment and reflects WHO’s overall health priorities for the biennium.^46^

**General and Fiduciary Funds**

WHO uses a method of fund accounting which segregates its resources into different categories or ‘funds’: the General Fund, the Special Purpose Fund, the Enterprise Fund and the Fiduciary Fund. Within the General Fund, there is an Assessed Contributions Fund (which manages ACs) and a Voluntary Fund (which manages VCs). The Fiduciary Fund ‘accounts for assets that are held by WHO in a trustee or agent capacity and that cannot be used to support the Organisation’s own programmes’.[Vaughan,^24^ p39]

For analysis of VCs, we drew on data from WHO’s audited financial statements and data provided in schedules 1 and 2 of the annex to the ‘Voluntary contributions by fund and by contributor’ reports, which are published yearly and available from WHO’s Financial Statements website.^27^ Schedule 1 provides disaggregated data on core, thematic and specified VCs, as well as six additional and separate categories of VCs: Stop TB (data available for biennia 2010–11 and 2012–13 only); Special Programme for Research and Training in Tropical Diseases; Special Programme of Research, Development and Research Training in Human Reproduction; Contingency Fund for Emergencies and Special Programmes and Collaborative Arrangements. During the period 2010–21, there have been revisions to the categories of VCs and their corresponding budget lines. For example, in 2014–15 a budget line ‘Contingency Fund for Emergencies’ appeared in the documentation, and from the biennium 2016–17, two new subcategories of specified VCs were introduced: Special Programmes and Collaborative Arrangements, and Outbreak and Crisis Response. Caution is therefore required when comparing VCs in reports when they were split across six categories with reports in which they were divided into eight categories. WHO documentation distinguishes between VCs to the General Fund, VCs to the Fiduciary Fund and non-cash VCs that cover ‘in-kind and in-service’ contributions. Fiduciary funds are ‘managed by WHO in a trustee capacity and cannot be applied in respect of the Organisation’s own programmes’.[WHO,^14^ p15] When referring to annual and biennial VCs, we are referring to VCs to the General Fund because VCs to the Fiduciary Fund do not fund WHO’s programmes but are passed on by WHO to other organisations (box 2). In the ‘Results’ section, we express VCs to the General Fund as a % of the final Programme Budget, VCs to the General Fund as a % of WHO’s total revenue, and VCs that are in-kind and in-service as a % of WHO’s total revenue. For both ACs and VCs, data were analysed using Microsoft Excel functions. All data were obtained between 1 March 2020 and 25 June 2022.

We disaggregate VCs according to the World Bank income groups, which categorise countries into four groups according to their gross national income (GNI): low-income, lower middle-income, upper middle-income and high-income.^28^ Additionally, we collate and present data on VCs, both monetary and non-monetary, from non-state actors. Donor classifications are informed by data from the UN and the World Bank.^28 29^ Donors are categorised as either: member state, other UN agency, other international organisation, international financial institution, partnership, philanthropy, non-government organisation/non-profit, private for-profit, and all other donors (which includes national parastatals, non-member states, private donors, subnational governments, universities and others that account for <1% each).

We acknowledge that a small number of donors contribute additional VCs *indirectly* to WHO by funding donors that contribute *directly* to the Organisation. For example, the Bill and Melinda Gates Foundation (BMGF) funds public-private partnerships such as Gavi the Vaccine Alliance, while member states fund other UN organisations such as United Nations Office for the Coordination of Humanitarian Affairs, which in turn funds WHO. A forensic analysis beyond the scope of this preliminary study would be required to accurately capture these indirect flows of VCs. When referring to quantities of VCs, we are referring to monetary value rather than the number of discrete voluntary contributions provided to WHO by donors. Regarding the latter, it is noteworthy that WHO signed 1300 separate agreements for programme budget VCs in 2021.

### Patient and public involvement

We did not involve patients or the public in any aspect of this research. They were not invited to contribute to its design, analysis or review of drafts of the manuscript.

## Results

**Figure 1:**
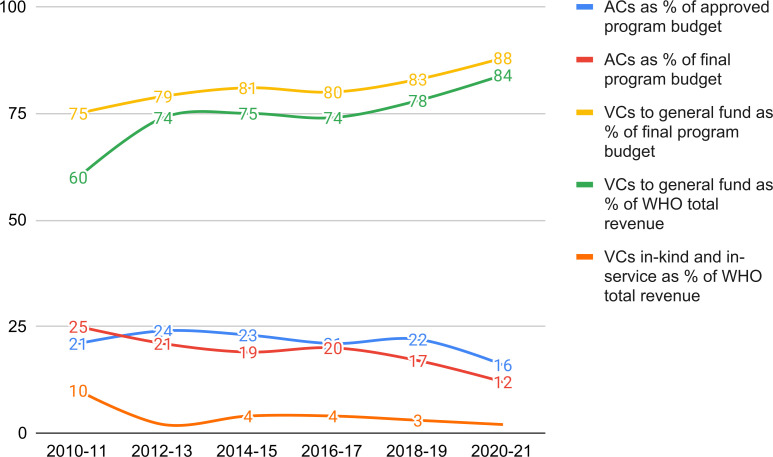
Assessed and voluntary contributions as per cent of approved and final Programme Budget, General Fund, and WHO total revenue. AC, assessed contribution; VC, voluntary contribution.

From 2010 to 2021, WHO’s budget has changed significantly in terms of flexibility, with VCs (which are mostly non-flexible) steadily increasing from 75% of WHO’s biennial Programme Budget in the 2010–11 biennium to 88% in 2020–21. The latter figure is 10% higher than the figure of four-fifths, which is widely cited by both the scholars and WHO.^1 6^ Conversely, ACs have fallen gradually, from 25% of WHO’s biennial Programme Budget in the 2010–11 biennium to 12% in 2020–21. As a percentage of WHO’s total revenue, VCs to the General Fund constituted 60% in 2010–11, increasing to 84% in 2020–21 (figure 1 and online supplemental file 1). With the exception of 2010–11, funding received from donors exceeded the Programme Budget approved at the WHA for each biennium. This is particularly noticeable for 2020–21 because of the COVID-19 pandemic.

**Figure 2:**
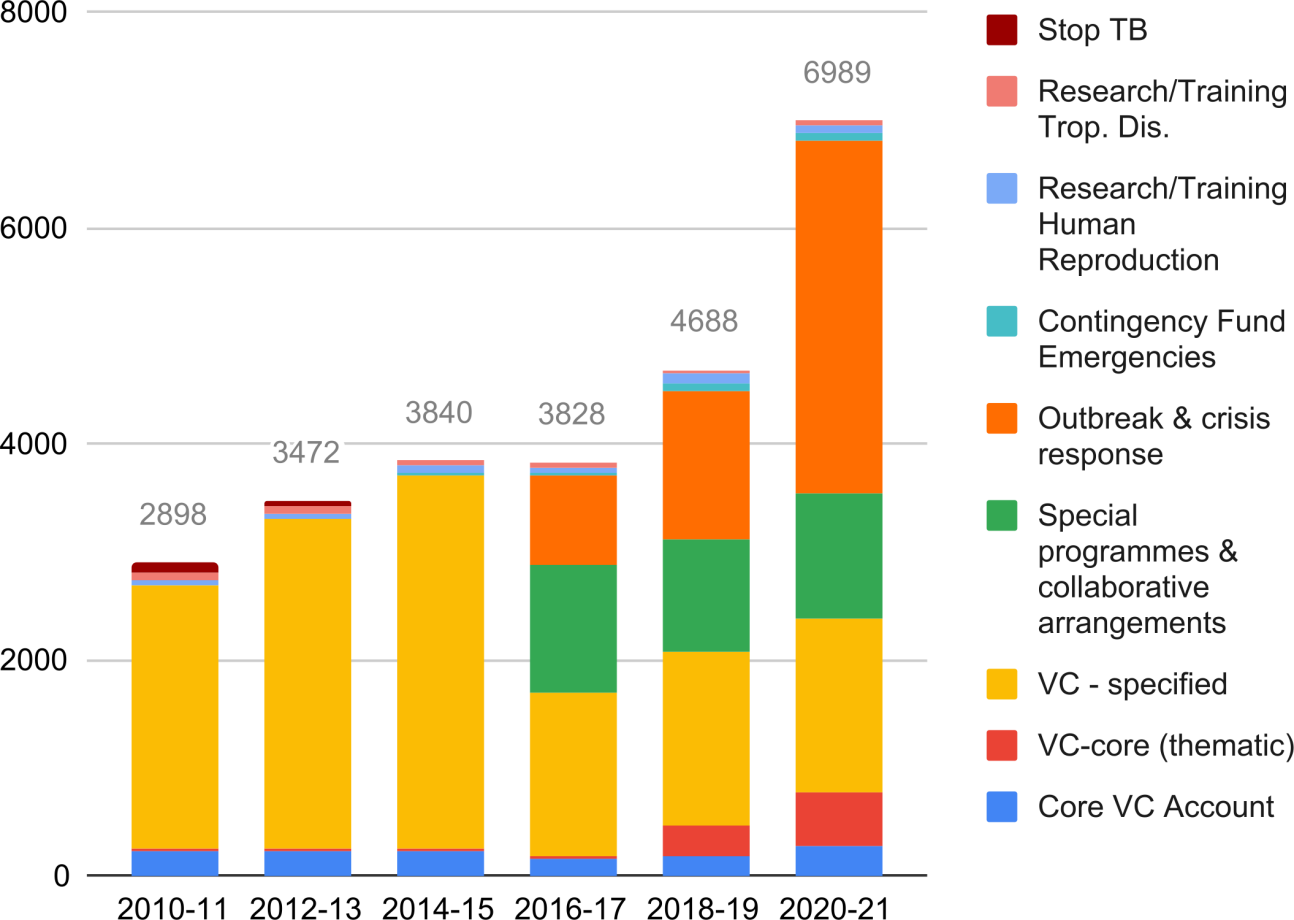
Voluntary contributions to the General Fund by sub-category per biennium. TB, tuberculosis; VC, voluntary contribution.

Figure 2 summarises all VCs to WHO’s General Fund. Given the caveat noted in the ‘Methods’ section with regard to how subcategories of VCs have changed over time, we provide a cautious estimate of specified VCs (least flexible) to the General Fund. Assuming that specified VCs are those VCs that are neither core nor thematic, then they have increased from US$2649 million to US$6224 million over the decade. In percentage terms, specified VCs constituted 91% of VCs to the General Fund in 2010–11 compared with 89% in 2020–21. Funding to the core VC category (most flexibility) increased slightly from US$235 million in 2010–11 to US$285 million in 2020–21, but there were stark undulations within this period, for example, core VCs dropped to US$148 million for 2016–17. For the thematic VCs (medium flexibility), there was a significant increase from US$14 million to US$480 million from 2010–11 to 2020–21.

**Figure 3:**
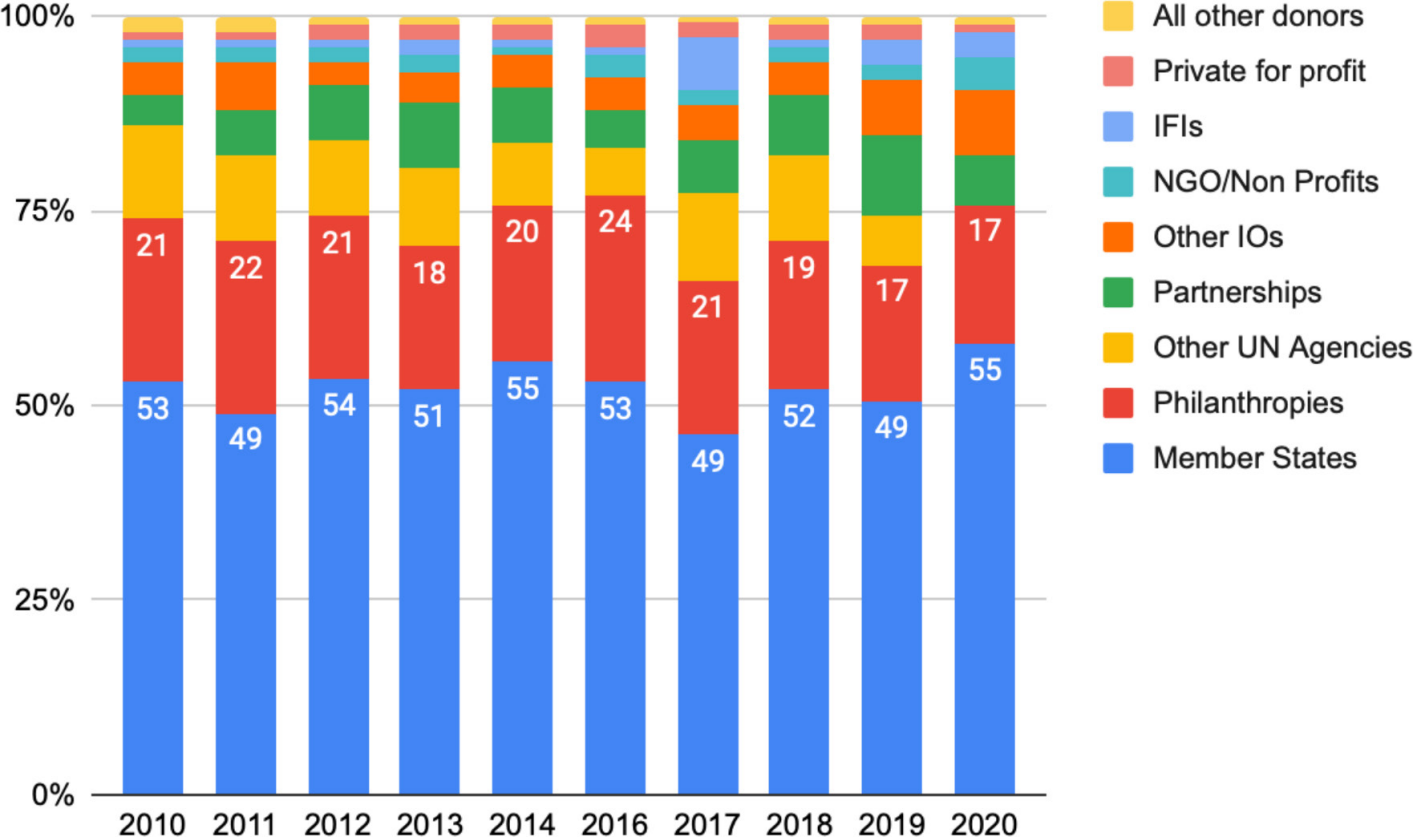
Voluntary contributions by donor type. NGO, non-governmental organisation; UN, United Nations; IFIs, International financial institutions; IOs, International Organizations.

There has been no change across the decade in the ranking of VCs by donor type, with member states contributing the most, followed by philanthropies, other UN agencies and partnerships (figure 3). Although the contributions from philanthropies remained significant, they account for a declining percentage of the total VCs to WHO, falling from a high of 24% in 2016 to 17% in 2020. The private-for-profit sector VCs to WHO have also steadily declined from a high of 3% in 2016 to 1% in 2020.

Across the period 2010–11 to 2020–21, in terms of monetary value the USA has contributed the most VCs to WHO, followed by BMGF and the UK. Over the 10 years period, the only VC donor not based in North America or Western Europe to make it into the top five is Japan. Since the COVID-19 pandemic, there have been some noticeable changes with Germany becoming the top VCs donor in 2020 and 2021 and and the UK falling outside the top 5 (online supplemental file 2).

10.1136/bmjgh-2022-011232.supp2 Supplementary data

Although pharmaceutical companies (and associated foundations) are not among the top 20 donors and their share of VCs are relatively small (about 1.7% of total VCs), their donations to WHO increased from US$18.1 million in 2012 to US$33.3 million in 2020. In 2016, this group collectively contributed US$51.8 million to WHO, which is greater than the combined VCs by member states from Central Asia, Eastern Europe, Latin America, South Asia and sub-Saharan Africa.

**Figure 4:**
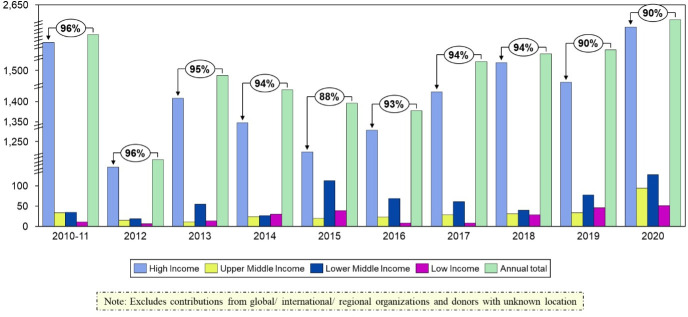
Voluntary contributions by donor country income group, 2010–20.

As one would expect, high-income countries (HIC) and donors based in HICs continued to provide a very high—although lower—percentage of total VCs: 90% in 2020 compared with 96% in 2010–11 (figure 4).

However, we found that the share of VCs to the General Fund provided by upper middle-income countries (UMICs) was consistently less than the share by lower middle-income countries (LMICs). Our findings show that LMICs accounted for almost twice the contributions of UMICs. To be clear, it is not because there are more LMICs than UMICs as there are an equal number (55 in 2020 and 54 in 2021) of countries in each category.^28^ Furthermore, we compared the share of global GNI of each income group with their share of VCs to WHO, to see if contributions were proportional to each group’s income. We found that the UMICs contributed the lowest proportion of their income to WHO of any of the four World Bank income groups (table 1).

**Table 1:** Per cent share of total voluntary contributions made by each income group vs per cent share of global GNI of each income group

Year		High-income	Upper middle-income	Lower middle-income	Low-income
2010–11	% share of total VCs	95%	2%	2%	1%
	% share of global GNI	68%	25%	6%	1%
2012	% share of total VCs	95%	2%	2%	1%
	% share of global GNI	66%	27%	6%	1%
2013	% share of total VCs	93%	1%	5%	1%
	% share of global GNI	65%	28%	6%	1%
2014	% share of total VCs	92%	2%	3%	3%
	% share of global GNI	65%	28%	7%	1%
2015	% share of total VCs	84%	2%	10%	4%
	% share of global GNI	65%	28%	7%	1%
2016	% share of total VCs	84%	2%	10%	4%
	% share of global GNI	65%	28%	7%	1%
2017	% share of total VCs	92%	3%	4%	1%
	% share of global GNI	64%	29%	7%	1%
2018	% share of total VCs	93%	2%	2%	2%
	% share of global GNI	64%	29%	7%	1%
2019	% share of total VCs	87%	3%	6%	4%
	% share of global GNI	63%	29%	7%	1%
2020	% share of total VCs	87%	3%	6%	4%
	% share of global GNI	64%	27%	9%	1%

Includes voluntary contributions received from member states only.

GNI, gross national income; VC, voluntary contribution.

## Discussion

Our discussion proceeds in three steps: first, we reflect on the structural defect underpinning WHO funding; second, we consider the incentives for donors to flexibly fund WHO; and finally, we suggest ways forward to further understand donors’ preference for voluntarily funding WHO.

Our study confirms the existence of a ‘structural defect’ underpinning WHO funding, understood in terms of inadequate financial support, limited control over funding and a reliance on a limited number of donors.^6^ While ACs remain at their mid-1990s levels because of the zero growth policies of the 1980s and 1990s,^2^ VCs have increased over the last decade from 75% to 88%. This is not what the architects of WHO intended at its inception. The intention was to create ‘a financial structure in which responsibility would be equitably distributed among all member states, taking into account each member’s relative wealth and population’, which would safeguard ‘both the agency’s autonomy and member states’ trust’.[M’ikanatha,^6^ p3] In an attempt to increase control over its funding, the Secretariat has sought to introduce and promote a range of flexible, voluntary funding streams for donors (box 1), but these are currently attracting very small amounts of funding. As we described in the ‘Introduction’ section, there are also reforms underway to mitigate the funding constraints faced by WHO. These should result in an increasing percentage of the base segment of WHO’s programme budget being funded by ACs but will not necessarily result in an increase in the total amount of funding to the base segment. Indeed, a freeze on the base segment of the programme budget for the 2024–25, announced at the Executive Board in February 2023, may temporarily cap the amount of funding in the near-term.^30^

In terms of reliance on a small number of donors, we found ‘unbalanced participation’ in funding to WHO.[M’ikanatha,^6^ p6] Over the past decade, HICs and foundations from HICs have dominated VCs to WHO, with the USA, the UK and BMGF consistently in the top three donors. Although the percentage share of monetary and in-kind VCs to WHO from philanthropies and for-profit donors has fallen since 2016, BMGF continues to contribute a very significant amount of money to WHO: the US$752 million in specified VCs for the biennium 2020–21—an increase of US$306 million from 2010 to 11—amounts to 13% of WHO’s approved 2020–21 Programme Budget.^31 32^ Germany has recently become the top VC donor to WHO, in part because of its explicit support for multilateralism and because of the suspension of US support during the Trump presidency.^33^ Tasked with the complex challenges of increasing the proportion of the base segment of the programme budget covered by flexible funding *and* also reducing reliance on a small pool of wealthy donors, member states have approved a recommendation of the WGSF to introduce a replenishment mechanism.^20^ Details of the mechanism are scant at the time of writing, and so we refrain from making a premature assessment. However, we are skeptical that such an innovation will resolve the challenge of ‘minilateralism’ (ie, reliance on a few donors to fund programmes), and also caution that it risks exacerbating ‘philanthrolateralism’ by outsourcing funding and decision-making to donors external to the UN system.^34^

Our analysis builds on previous studies by identifying an inequitable distribution of member state VCs to WHO: LMICs contributed twice as much total VCs as UMICs. It is reasonable to ask why this is the case? The scholarly literature tends to focus on HICs and the negative consequences of their earmarked funding for multilateralism. One study describes the insidious effects of earmarking as ‘trojan multilateralism’ whereby a few wealthy donors seek to inculcate their goals and interests into an organisation such as WHO under the guise of multilateral support.^12^ On the one hand, our results show ‘trojan multilateralism’ in evidence at WHO with the dominance of VC funding coming from a few wealthy donors; on the other, our analysis suggests little—if any—support for trojan multilateralism among UMICs. Given the very high levels of VCs from a few HICs and BMGF, UMICs would need to increase their VCs significantly in order to effect change within WHO. Conversely, the relatively high VCs from LMICs may reflect a concerted effort by this group of countries to counter the influence within WHO from HICs. Despite efforts by WHO Secretariat to promote the idea of global public goods and the universal benefits of fully funded health programmes, those benefits would most likely fall on health systems within LMICs. It is no coincidence that LMICs and the poorer UMICs direct the majority of their VCs to fund the Outbreak and Crisis Response and the Contingency Fund for Emergencies budget lines of the General Fund, both of which have seen significant increases in voluntary funding during the pandemic.^35^ There is a further incentive, therefore, for these countries to direct their voluntary donations to programmes that would otherwise remain underfunded.

A common response from global health scholars has been repeated calls to simply increase the total amount of ACs to WHO. Following the Ebola epidemic in 2014, Gostin called for a doubling of WHO’s budget within 5 years and for 50% of the total to come from ACs.^36^ During COVID-19, M’ikanatha and Welliver argued that it would be ‘realistic’ for WHO’s budget to triple, with at least 70% of the total provided by ACs ‘if there is political will’.[M’ikanatha,^6^ p8] Unfortunately, the extent of member states’ political will has not matched such high expectations, as evidenced by the recommendations of the WGSF final report: an increase in ACs from 22% to 50% of the base segment of the 2022–23 Programme Budget by 2028–29.^37^ In monetary terms, this is an increase in ACs from US$957 million to US$2182 million for 2028–29.^38^

Described as the ‘lifeblood’ of WHO, the benefits of ACs are well-understood: they provide long-term, predictable financing, are ‘uniquely—fully flexible, and can be allocated to any type of work’, and unlike VCs they can be allocated ‘throughout the biennium in a strategic and timely manner to ensure alignment of funding across the programme budget in its entirety’.[WHO,^39^ p12; Seitz,^34^ p11] Currently, as our analysis shows, all of the advantages of ACs thus described relate to just 12% of WHO’s budget. But with little prospect of an increase in total ACs, how else might WHO be flexibly funded? In his opening speech to the WGSF at its final meeting in April 2022, the WHO DG remarked: “I realise there remains a spectrum of opinion on how best to reset the course of WHO’s financing, with its dependence for more than 80 percent of its budget on voluntary, mostly earmarked, contributions. But there is agreement that the current system is not fit for purpose”.^40^ The way forward is to correctly identify what it is about the ‘current system’ that is ‘not fit for purpose’.

It is remarkable that very few peer-reviewed studies have interviewed donors and WHO officials to better understand both the implications for and drivers of VCs.^10 11^ While it is the case that WHO regularly consults with and seeks member state and stakeholders’ views, these consultations are internal processes and the results are rarely published. From interviews with just 20 current and former WHO staff, Daugirdas and Birci gained crucial insights into donor practice, including a preference for ‘concrete, measurable outcomes’ that satisfied definitions of development approved by the Organisation for Economic Cooperation and Development’s Development Assistance Committee.[Daugirdas,^10^ p319] Conceptual analysis supports the need to focus on donor behaviour and preferences. Bauman, for example, contends that in order to break the vicious cycle of earmarking funds within the UN system, an understanding of the dynamics of collective action, the social construction of norms and the influence of bureaucratic structures in incentivising certain behaviours is necessary.^9^

The WGSF provided a welcome opportunity for donors to reflect on the core functions of WHO and to decide how best to fund them. Its recommendations include ‘boosting’ funding to WHO that is flexible, sustainable and predictable, and it has secured approval for a modest increase in ACs to the base segment of WHO’s Programme Budget. However, as Baumann argues: ‘Rather than ensuring a minimum level of core [funding], it might be more important to avoid a toxic level of non-core that triggers systemic change’.[Baumann,^9^ p356] Unfortunately, the WGSF did exactly what Baumann warned against: it focused on securing core funding from additional ACs when the more important challenge is ‘the need to address the incentives that come with earmarked funding’.[Baumann,^9^ p356] With the adoption at WHA75 of the recommendations of the WGSF, the Task Group will continue the work of the WGSF by providing ‘long-term improvements…within the mandate identified by the recommendations of (WGSF)’.[WHO,^41^ p2] The Task Group presented a report to the Executive Board in February 2023, which describes only the ‘management’ of VCs expressed in terms of ‘efficiency gains’[WHO,^41^ p6]. We argue that this managerial focus will not address the key insights provided by Baumann. Further enquiry is required to understand why member states contribute VCs to WHO. We suggest that this is work that could and should be conducted by the Task Group.

Our analysis of VCs reveals a number of inefficiencies at the core of WHO’s funding model. It is ironic that member states repeatedly extoll the virtues of WHO’s normative role (most recently during discussion at the 152nd Executive Board) and yet this core function remains chronically underfunded. Daugirdas and Birci point out that VCs also come with a significant administrative burden for WHO staff, consuming valuable time and resources. Furthermore, WHO staff are required to spend an increasing amount of time nurturing and managing relationships with potential donors.^13 42^ Nevertheless, member states repeatedly criticise WHO’s Secretariat for allocating too high a proportion of the base segment to cover staff costs, 95% of which are funded by ACs because VCs are too unreliable and unpredictable. Finally, calls from WHO for additional funding are met with requests from donors for WHO to be more efficient with its resources, overlooking that fact that WHO’s increasing reliance on VCs require it to expend significant resources managing ‘discrete health issues…pursued through short-term initiatives’[Reddy,^2^ p8]—the polio transition, for example. A recent comment in *Nature* offers this thinly veiled rebuke: ‘One way to increase efficiency would be to relieve the agency of the need to devote so many of its resources to the priorities of individual governments’.[Editorial,^43^ p7]

There are a number of limitations to our study. We reiterate that caution is required when comparing VCs across different biennia. We offer a quantitative analysis of VCs that relies on funding data published by WHO. As we discuss, and recommend, qualitative research into donor preferences for VCs would provide nuance to our work. Finally, while our analysis focuses on GNI, we recognise that GNI does not necessarily reflect the economic realities of the population or income distribution within each country.

Nevertheless, peer-reviewed analysis of WHO funding remains scant and our analysis adds to this literature by suggesting various novel entry points for further study. Norms imply patterns of behaviour that are mutually constituted rather than fixed, and thus subject to change. Further research that seeks to better understand donor incentives for earmarked funding of WHO would therefore be welcome. According to the Chair of WHO’s Independent Expert Oversight Advisory Committee, ‘WHO was one of the most transparent organisations with which he has worked, as data and information are readily and publicly available’.[WHO,^44^ p1] From our perspective as researchers, we found that while the data were accessible, they were not easy to collate or interpret and, year-on-year, require detailed and sustained monitoring. The establishment of a dedicated research centre (either within WHO or independent of it) could provide resources necessary to illuminate for the lay person what are often opaque funding decisions at WHO. The organisation’s funding is a politically sensitive area for research and access to economists at WHO working to develop its budgets and financial statements is not possible. We would encourage WHO to embrace a more open research environment between its staff and independent researchers.

## Conclusion

WHO remains constrained by the conditions attached to the vast majority of funding that it receives from its donors. The most flexible funding—ACs from member states—has fallen from 25% of the revenue to WHO’s Programme Budget in 2010–11 to 12% in 2020–21. Conversely, VCs, which vary in their flexibility but are much less flexible than ACs, have risen from 75% to 88% of the revenue to WHO’s Programme Budget in the same period.

HICs and philanthropies based in those countries contributed the most VCs to WHO (90% in 2020). LMICs, however, contributed twice as much in VCs than UMICs. Relative to their GNI, LMICs are contributing far more. This indicates that UMICs are not ‘pulling their weight’ with regard to funding WHO.

In May 2022, member states approved the WGSF’s recommendation to increase the amount of ACs to the base segment of WHO’s Programme Budget. This is welcome news even though it will result in only a modest increase in flexible funding. We also support the WGSF’s call for all VCs to the base segment of the Programme Budget to be fully flexible and encourage the WHO Secretariat and member states to ‘boost’ their commitment to flexible funding. The work to ensure a flexibly funded WHO must continue by the Agile Task Group, and we argue that it should broaden its focus on VCs to include research to better understand why donors prefer to voluntarily fund WHO. We suggest that this may be a more effective way to resolve the structural defect of WHO’s funding model than periodically calling for more ACs.

## Data Availability

All data relevant to the study are included in the article or uploaded as supplementary information.
