# Supplementary material for: Flexibly funding WHO? An analysis of its donors’ voluntary contributions
Source: BMJ Glob Health. 2023 Apr 6;8(4):e011232. doi: 10.1136/bmjgh-2022-011232 (PMC10083790; doi:10.1136/bmjgh-2022-011232)
Supplement: Supplementary data [file bmjgh-2022-011232supp001.pdf]

Supplemental file 1: Biennial WHO Funding Data, 2020-11 to 2020-21

$m*	2010-11	2012-13	2014-15	2016-17	2018-19	2020-21
**AC approved**	945	949	929	929	957	957
**VC General Fund**	2899	3469**	3839	3828	4687	6989
**VC Fiduciary Fund**	174	187	59	66	103	89
**VC in-kind + in-service**	488	110	180	225	152	173
**Program budget approved**	4540	3959	3977	4385	4422	5840
**Program budget total revenue**	3844	4418	4794	4755	5678	8004
**WHO total revenue*** **	4848	4675	5097	5139	6017	8365

* Figures are rounded up, which accounts for small discrepancies with Fig 2.
** Elimination of inter-fund transfers = $3m, which accounts for discrepancy with Fig 2.
*** In addition to ACs and VCs from donors, additional sources of revenue to WHO include: beneficial exchange rates, rental income and fees for services.
