# Supplementary material for: Flexibly funding WHO? An analysis of its donors’ voluntary contributions
Source: BMJ Glob Health. 2023 Apr 6;8(4):e011232. doi: 10.1136/bmjgh-2022-011232 (PMC10083790; doi:10.1136/bmjgh-2022-011232)
Supplement: Supplementary data [file bmjgh-2022-011232supp002.pdf]

**Supplemental file 2: Donors ranked by the total value of VCs provided annually to WHO for the period 2010-21 (donor contribution as % of total value of VCs).**

| | 2010-11 | 2012 | 2013 | 2014 | 2015 | 2016 | 2017 | 2018 | 2019 | 2020 | 2021 |
|---|---|---|---|---|---|---|---|---|---|---|---|
| **1** | BMGF (15.2%) | BMGF (17.0%) | BMGF (15.6%) | USA (14.9%) | USA (16.4%) | USA (18.6%) | USA (18.9%) | USA (12.5%) | USA (13.7) | Germany (16.5%) | Germany (18.2%) |
| **2** | USA (15%) | USA (13.7%) | UK (14.0%) | BMGF (12.8%) | UK (10.5%) | BMGF (15.8%) | BMGF (15.3%) | BMGF (10.1%) | Gavi (9.4%) | BMGF (10.3%) | BMGF (11.3%) |
| **3** | UK (9.9%) | UK (7.4%) | USA (9.3%) | UK (7.7%) | BMGF (9.9%) | UK (7.7%) | UK (7.7%) | UK (9.1%) | BMGF (9.3%) | UK (7.2%) | US (10.6%) |
| **4** | Canada (5.3%) | Canada (6.1%) | Gavi (6.7%) | Gavi (6.4%) | Gavi (6.8%) | Gavi (4.3%) | World Bank (6.9%) | Gavi (7.0%) | UK (8.8%) | EC (6.7%) | Gavi (7.3) |
| **5** | Rotary (4.0%) | Gavi (5.9%) | Canada (6.3%) | Canada (4.0%) | NPT (4.7%) | Japan (3.9%) | Gavi (6.3%) | Germany (6.8%) | EC (5.7% | Gavi (5.1%) | EC (6.7%) |
| **6** | Norway (3.9%) | Australia (4.2%) | Norway (3.5%) | Rotary (3.3%) | Nigeria (3.6%) | NPT (3.8%) | Germany (4.2%) | UNOCHA (6.0%) | Germany (5.6%) | Saudi Arabia (3.9%) | UK (5.4%) |

EC = European Commission; NPT = National Philanthropic Trust.
NB: The annex to audited financial statements provides annual rather than biennial VC data.
